# Mortality in Children Aged 0-9 Years: A Nationwide Cohort Study from Three Nordic Countries

**DOI:** 10.1371/journal.pone.0146669

**Published:** 2016-01-08

**Authors:** Yongfu Yu, Guoyou Qin, Sven Cnattingius, Mika Gissler, Jørn Olsen, Naiqing Zhao, Jiong Li

**Affiliations:** 1 Section for Epidemiology, Department of Public Health, Aarhus University, Aarhus, Denmark; 2 Department of Biostatistics, School of Public Health, Fudan University, Shanghai, China; 3 Clinical Epidemiology Unit, Department of Medicine Solna, Karolinska University Hospital, Karolinska Institute, Stockholm, Sweden; 4 Information Department, THL National Institute for Health and Welfare, Helsinki, Finland; 5 Department of Epidemiology, School of Public Health, University of California Los Angeles, Los Angeles, California, United States of America; 6 Department of Clinical Epidemiology, Aarhus University Hospital, Aarhus, Denmark; Rutgers University, UNITED STATES

## Abstract

**Background:**

Mortality in children under five years has been widely studied, whereas mortality at 5–9 years has received little attention. Using unique data from national registers in three Nordic countries, we aimed to characterize mortality directionality in children aged 0 to 9 years.

**Methods and Findings:**

The cohort study included all children born in Denmark from 1973 to 2008 (n = 2,433,758), Sweden from 1973 to 2006 (n = 3,400,212), and a random sample of 89.3% of children born in Finland from 1987 to 2007 (n = 1,272,083). Children were followed from 0 to 9 years, and cumulative mortality and mortality rates were compared by age, gender, cause of death, and calendar periods. Among the 7,105,962 children, there were 48,299 deaths during study period. From 1981–1985 to 2001–2005, all-cause mortality rates were reduced by between 34% and 62% at different ages. Overall mortality rate ratio between boys and girls decreased from 1.25 to 1.21 with the most prominent reduction in children aged 5–9 years (from 1.59 to 1.19). Neoplasms, diseases of the nervous system and transport accidents were the most frequent cause of death after the first year of life. These three leading causes of death declined by 42% (from 6.2 to 3.6 per 100,000 person years), 43% (from 3.7 to 2.1) and 62% (from 3.9 to 1.5) in boys, and 25% (from 4.1 to 3.1 per 100000 person years), 42% (from 3.4 to 1.9) and 63% (from 3.0 to 1.1) in girls, respectively. Mortality from neoplasms was the highest in each age except infants when comparing cause-specific mortality, and half of deaths from diseases of the nervous system occurred in infancy. Mortality rate due to transport accidents increased with age and was highest in boys aged 5–9 years.

**Conclusions:**

Mortality rate in children aged 0–9 years has been decreasing with diminished difference between genders over the past decades. Our results suggest the importance of further research on mortality by causes of neoplasms, and causes of transport accidents—especially in children aged 5–9 years.

## Introduction

The global effort towards Millennium Development Goal 4 has led to reduced mortality rate in children younger than 5 years, from 90 deaths per 1,000 live births in 1990 to 48 deaths in 2012. With the exception of Sub-Saharan Africa and Oceania, mortality has at least halved in most regions [1]. As a consequence, an estimated additional 90 million children survived their 5^th^ birthday during the past two decades, leading to an increase of population beyond 5 years [2]. Mortality under five years has been widely studied, and the need to focus on the health and development of adolescence has been emphasized by the United Nations [3]. In contrast, health and mortality in children aged 5 to 9 years have been almost neglected, even with a population of 600 million worldwide.

The scarcity of detailed data has been an obstacle to gain a basic understanding of mortality directionality over time in age, gender, and cause of death among children from 0 to 9 years. In previous childhood mortality studies, age-specific mortality rates are not known since children aged 0–9 years are typically merged in even larger aggregated age-groups, like 0–14 years [4], or 1 month to 15 years [5]. Based on unique data from national registers in three Nordic countries, we aimed to characterize mortality in children aged 0 to 9 years, according to age, calendar time, gender, and cause of death.

## Materials and Methods

### Study design

This population-based cohort study used nationwide data from Denmark, Sweden and Finland. All live-born and new residents in these three Nordic countries were assigned a unique civil personal identification number, which permits accurate individual information linkage across different national registries [6]. We included all children born in Denmark from 1973 to 2008 (n = 2,433,758) and in Sweden from 1973 to 2006 (n = 3,400,212), and a random sample of 89.3% of children born in Finland from 1987 to 2007 (n = 1,272,083) following strict data protection rules in Finland, because the authorities in Finland do not allow to extract 100% complete data from the targeted population. Follow-up started at birth and ended at one of the following events, which ever came first: death, emigration, the day before the 10^th^ birthday or end of follow-up (The follow-up ended until December 31^st^ 2009 in Denmark, December 31^st^ 2008 in Sweden and December 31^st^ 2010 in Finland). We excluded 91 children with no information on gender.

### Outcomes

The main outcomes of interest were all-cause mortality rate, age-, gender- and cause-specific mortality rate, and type of death (natural death from diseases or medical conditions, unnatural death from external causes of injuries and poisonings) mortality rate in children aged 0 to 9 years. We obtained information on cause of death from the Cause of Death Registers in the three countries. The death statistics are based on individual data in death certificates and contains the date of death and cause of death of individuals. In Denmark, the International Classification of Disease (ICD) 8th version was used to classify the cause of death before 1994 and ICD-10 was used since 1994; in Sweden, ICD-8 was used from 1973 to 1986, ICD-9 from 1987 to 1996 and ICD-10 was used since 1997; and in Finland, ICD-9 was used from 1987 to 1995 and ICD-10 was used since 1996. For Finnish data, we do not have detailed ICD Codes and death by the underlying cause of death was divided into 54 main groups based on ICD codes[7]. Type of death was separated into two categories: natural death from diseases or medical conditions (ICD-8 codes 000–799, ICD-9 codes 000–799, and ICD-10 codes A00-R99), and unnatural death from external causes of injuries and poisoning (ICD-8 codes E800-E999, ICD-9 codes E800-E999, andICD-10 codes V01-Y98). Natural death were classified into the following main groups according to the ICD system: infections and parasitic diseases (ICD-8 and ICD-9 codes 000–1399, ICD-10 codes A00–B999); neoplasms (ICD-8 and ICD-9 codes 140–2389, ICD-10 codes C00–C999); endocrine, nutritional, and metabolic diseases (ICD-8 and ICD-9 codes 240–2799, ICD-10 codes E00–E909); diseases of the nervous system and the sense organs (ICD-8 and ICD-9 codes 320–389, ICD-10 codes G00–H95); diseases of the circulatory system (ICD-8 and ICD-9 codes 410–414, 420–423,425–429, ICD-10 codes I20–I25, I30–I33, I39–I52); diseases of the respiratory system (ICD-8 and ICD-9 codes 460–519, ICD-10 codes J00–J99); diseases of the digestive system (ICD-8 and ICD-9 codes 520–579, 4442, ICD-10 codes K00–K93); certain conditions originating in the prenatal periods(ICD-8 and ICD-9 codes 760–779, ICD-10 codes P00–P96); congenital malformations (ICD-8 and ICD-9 codes 740–759, ICD-10 codes Q00–Q99); sudden infant death syndrome (ICD-9 codes 798.0, ICD-10 codes R95), unknown or unspecified causes (ICD-8codes 795–796, and ICD-9 codes 798.1–9,799 and ICD-10 codes R96-R99); and other diseases. Unnatural death were classified into the following main groups: transport accidents(ICD-8 and ICD9 codes 800–848, ICD-10 codes V01–V99); drowning (ICD-8 and ICD-9 codes 910, ICD-10 codes W65–W74); other external cause (ICD-8 and ICD9 codes 849–900 except 910, ICD-10 codes W00-Y89 except W65-W74).

### Statistical Analysis

Follow-up time was counted by years and time at risk was calculated for each child. Mortality rate was calculated by dividing the number of deaths by the person time at risk and expressed as the number of deaths per 100,000 person-years (PY) at risk. Rate ratio was calculated to compare the risk of death between different groups [8]. Cumulative mortality measured the probability of death before a given age. Children aged 0–9 were categorized into ten groups at one-year interval, while infant deaths (<1 year) were subdivided into neonatal (0–27 completed days) and post-neonatal (28–364 days) mortality. To analyze the change of mortality over the period, the follow-up calendar time was grouped into seven periods (1973–1980, 1981–1985, 1986–1990, 1991–1995, 1996–2000, 2001–2005, and 2006–2010). Mortality was estimated according to all-cause mortality, mortality by type of death (natural vs. unnatural cause of death) and cause-specific mortality, stratified by age, gender and calendar period. Other deaths were considered as competing events while calculating cause-specific mortality. In order to compare with previous studies, we calculated all-cause mortality not only in each age group but also in three aggregated age groups (<1 year, 1–4 and 5–9 years). Due to the small numbers of death, mortality rate was only observed in the three aggregated age groups when analyzing cause-specific mortality. As the death information during 1973–1980 and 2006–2010 was incomplete in five-year interval, we defined mortality in 1981–1985 as baseline and 2001–2005 as current level to assess the degree of mortality change over time except for special notice. As we did not have detailed ICD codes of Finland, only data from Denmark and Sweden were used when calculating some specific causes of death (Table 1). Analysis was conducted in SAS software (SAS 9.2, SAS Institute Inc., Cary, North Carolina, USA).

**Table 1:** The number of deaths by causes of death in aggregated groups.

	Denmark &Sweden			Finland			Denmark, Sweden & Finland			
Cause of death	<1y	1-4y	5-9y	<1y	1-4y	5-9y	<1y	1-4y	5-9y	Total
Unnatural death from diseases or medical conditions										
Infectious and parasitic disease	754	393	81	65	52	14	819	445	95	1,359
*Meningococcal infection*^£^	*94*	*187*	*33*	*-*	*-*	*-*	*94*	*187*	*33*	*314*
Neoplasms	306	949	857	63	151	147	369	1,100	1,004	2,473
*Leukemia*^¶^ ^£^	*71*	*314*	*277*	-	-	-	*71*	*314*	*277*	*662*
*Brain and other central nervous system tumours*^¶^ ^£^	*77*	*315*	*282*	-	-	-	*77*	*315*	*282*	*674*
*other causes of neoplams*^¶^ ^£^	*158*	*320*	*298*	-	-	-	*158*	*320*	*298*	*776*
Endocrine, nutritional and metabolic diseases	411	268	101	97	42	38	508	310	139	957
Diseases of nervous system	855	551	213	98	66	33	953	617	246	1,816
*Meningitis*^¶^ ^£^	*299*	*143*	*20*	-	-	-	*299*	*143*	*20*	*462*
*Cerebral palsy*^¶^ ^£^	*91*	*72*	*43*	-	-	-	*91*	*72*	*43*	*206*
*Other causes of diseases of nervous disease*^¶^ ^£^	*465*	*336*	*150*	-	-	-	*465*	*336*	*150*	*951*
Diseases of circulatory system	167	104	56	44	31	18	211	135	74	420
Diseases of respiratory system	749	327	75	62	39	13	811	366	88	1,265
*Pneumonia*^¶^ ^£^	*500*	*129*	*26*	-	-	-	*500*	*129*	*26*	*655*
Diseases of the digestive system	232	84	25	12	8	3	244	92	28	364
Certain conditions originating in the perinatal period^¶^	13,582	78	23	-	-	-	13,582	78	23	13,683
Congenital malformations	10,937	1,309	329	1,700	189	65	12,637	1,498	394	14,529
Sudden infant death syndrome^¶^	1,327	7	-	-	-	-	1,327	7	-	1,334
Unknown or unspecified causes	2,479	152	38	22	11	3	2,501	163	41	2,705
Other diseases	390	322	248	2,496	52	16	2,886	374	264	3,524
Unnatural death from external causes of injury and poisoning	-	-	-	-	-	-	-	-	-	-
Transport accidents	111	490	582	22	80	98	133	570	680	1,383
Drowning	18	314	184	4	93	51	22	407	235	664
Other external causes	380	751	408	89	112	83	469	863	491	1,823

¶: ICD codes only available in Denmark and Sweden

£: subgroup within main groups

## Results

A total of 7,105,962 children were followed up for 62,649,303 person-years. During the period, 147,658 children (2%) were censored due to emigration. A total of 48,299 deaths were identified and 343 had no information on causes of death (0.67%). Certain conditions originating in the prenatal periods, congenital malformations, sudden infant death syndrome, and unknown or unspecified causes were the most important causes of death in infancy (Table 1 and S1A Table). Although the majority of death by congenital malformations happened in infancy, congenital malformations were still the important cause of death in children between 1 and 9 years. After 1 year of age, neoplasms, diseases of the nervous system and transport accidents were the most frequent cause of death (Table 1 and S1A Table). Overall cumulative mortality before 10 years was 696 (95% CI: 690–702) per 100,000, for boys 777 (95% CI: 767–786) per 100,000 and for girls 611 (95% CI: 602–619) per 100,000.

### All-cause mortality, infant mortality, time directionality and difference between genders

**Fig 1:**
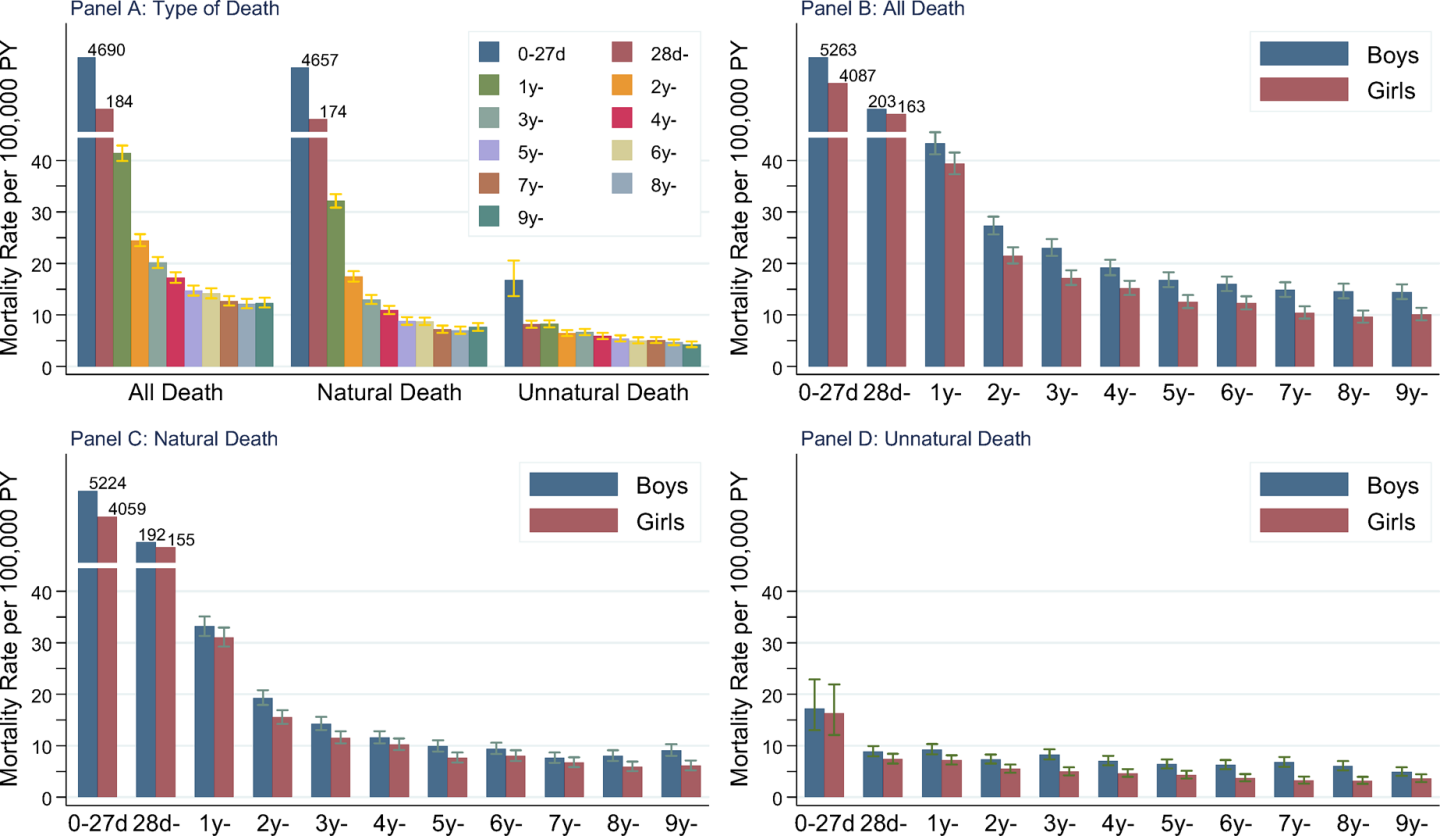
Mortality rates in children aged 0–9 years over type of death and age (28d: 28–364 Days).

All-cause mortality rate in children aged 0–9 years decreased with age and differed slightly between 5 and 9 years. The directionality was similar in both genders (Fig 1A). In the entire study period, mortality rate before 10 years was 86.1 per 100,000 person years in boys and 67.7 per 100,000 person-years in girls, giving a rate ratio of boys to girls (RR_m/f_) of 1.27 (95% CI: 1.25–1.29). All-cause mortality rate was higher in boys than that in girls according to age, but gender difference was more pronounced in children aged 5–9 years than that in younger children (Fig 1B). In almost all ages between 5 and 9 years, RR_m/f_ were greater than 1.30, whereas in children younger than 5 years, all RR_m/f_ were lower than 1.30. Regarding aggregated age groups, mortality difference between genders was more pronounced in children aged 5–9 years (RR_m/f_: 1.39, 95% CI: 1.31–1.49) compared to the other two aggregated age groups (<1 year, 1.27 [95% CI: 1.25–1.30]; 1 to 4 years, 1.21 [95% CI: 1.15–1.27]). Similar results were observed for natural death from diseases or medical conditions and unnatural death from external causes of injuries and poisonings (Fig 1C and 1D).

**Fig 2:**
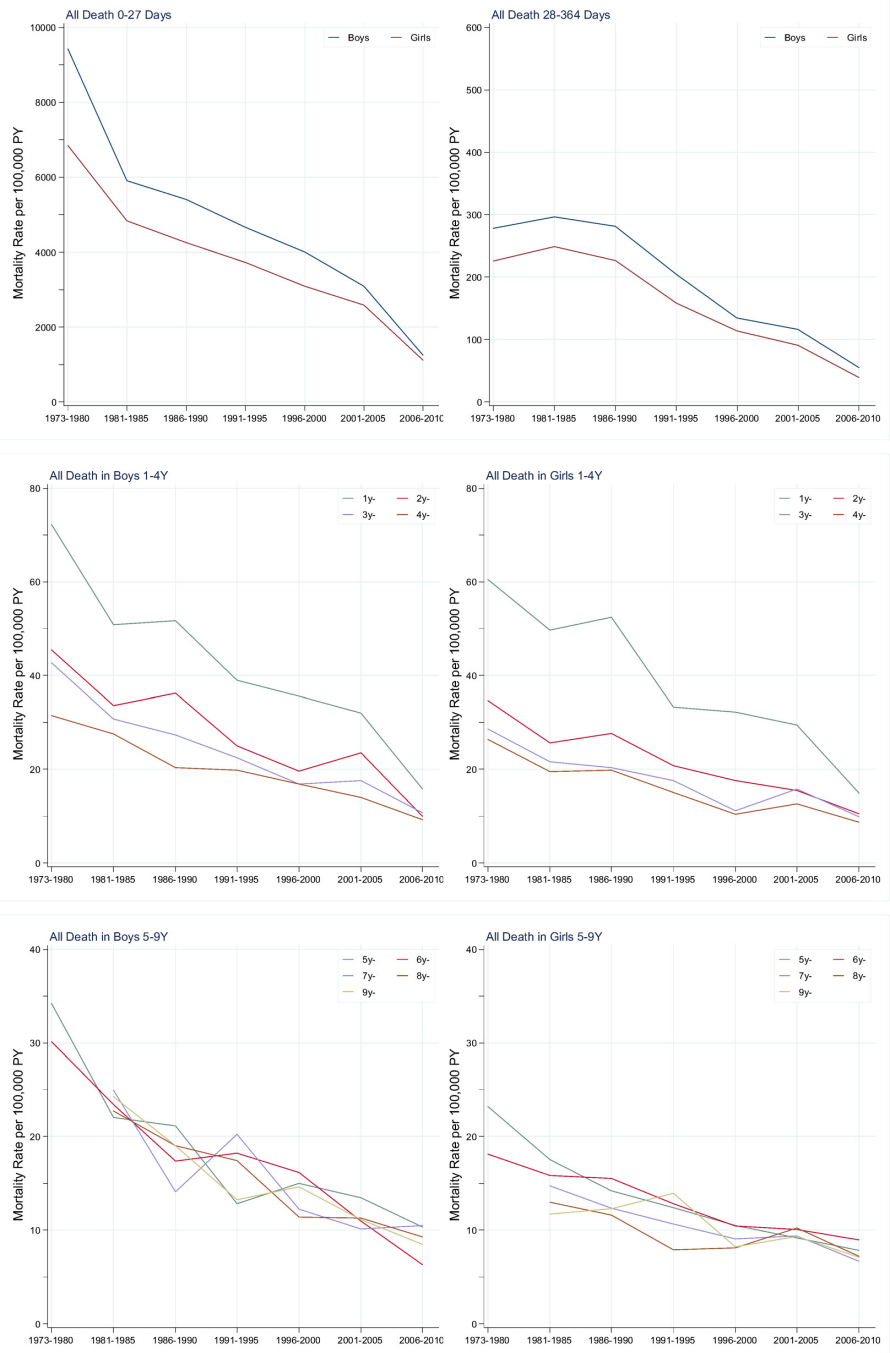
All-cause mortality rates in children aged 0–9 years over calendar periods and age.

When taking length of follow-up time into account, all-cause mortality rates in children aged 0–9 years decreased with calendar periods for a given age, and dropped with age for a given calendar period (Table 2). Compared to the period of 1981–1985, mortality rates in the period of 2001–2005 were decreased by more than 40% in most age groups, ranging from a decline of 34% to 62%. The reduction in infant mortality was 52%, and the reductions in neonatal and post-neonatal mortality were 47% and 62%, respectively. An estimate of 87.0% of death due to congenital malformations, 92.8% of death due to unknown or unspecified causes and 99.3% of death due to certain conditions originating in the prenatal periods and happened in infancy. From 1981–1985 to 2001–2005, mortality rate due to certain conditions originating in the prenatal periods, unknown or unspecified causes and congenital malformations decreased by 33%, 94% and 58% (34%, 95% and 59% in boys, 31%, 93% and 56% in girls), respectively. Because the ICD codes of sudden infant death syndrome began from 1987 in our database, we used the mortality rate in 1991–1995 as baseline to assess mortality change over time. Mortality rate due to sudden infant death syndrome (SIDS) decreased by 47% (54% in boys, 35% in girls) from 1991–1995 to 2001–2005. It should be noted that infant mortality rate, accounting for 70% of the total deaths, still remained dramatically higher than other age-related mortality rates (1–9 years) during the recent years of 2006–2010 (Table 2). Both natural death and unnatural death had similar results (S2 Table and S3 Table). In both genders, we found a similar pattern of all-cause mortality rates over calendar periods and age (Fig 2; S1B Table). During 1981–1985, boys had higher mortality rates than girls in all age groups; mortality rates in boys and girls were 97.45 and 77.94 per 100,000 person years, respectively. Mortality rates in boys were higher than in girls, but the gender difference in mortality rate began to decrease in 1996–2000. During 2001–2005, mortality rates in boys and girls were 48.38 and 39.97 per 100,000 person years, respectively. RR_m/f_ in children less than 10 years decreased from 1.25 (95% CI: 0.93–1.68) to 1.21 (95% CI: 0.80–1.84) with the most prominent reduction in children aged 5–9 years (from 1.59 [95% CI: 0.83–3.06] to 1.18 [95% CI: 0.5–2.78]). However, statistically significant gender differences were only observed in children under 1 year with RR_m/f_ of 1.21 (95% CI: 1.09–1.35) in 1981–1985 and 1.22 (95% CI: 1.04–1.43) in 2001–2005 (S4 Table).

**Table 2:** Total mortality rates per 100,000 person years by age and calendar period.

Age		1973–1980	1981–1985	1986–1990	1991–1995	1996–2000	2001–2005	2006–2010	d%^a^
0–27 days	IR(95%CI)	8,167.0(7994.2–8343.5)	5,382.6(5196.6–5575.3)	4,843.7(4699.0–4993.0)	4,204.4(4077.1–4335.7)	3,561.4(3435.4–3692.1)	2,841.1(2729.4–2957.5)	1,185.7(1073.4–1309.8)	47%
	Cases/PY	8,400/102,853	3,106/57,704	4,171/86,111	4,062/96,613	2,959/83,084	2,385/83,945	388/32,723	
28–364 days	IR(95%CI)	252.6(243.6–261.9)	273.1(261.1–285.7)	254.7(244.9–264.8)	181.8(174.2–189.7)	124.1(117.4–131.2)	104.0(97.9–110.5)	47.2(41.5–53.7)	62%
	Cases/PY	2,940/1,163,932	1,889/691,628	2,517/988,237	2,126/1,169,412	1,251/1,008,036	1,044/1,004,074	234/495,667	
1y-	IR(95%CI)	66.5(61.9–71.5)	50.3(45.5–55.6)	52.0(47.7–56.8)	36.2(33.0–39.7)	34.0(30.7–37.6)	30.8(27.6–34.3)	15.4(12.7–18.5)	39%
	Cases/PY	734/1,103,580	378/751,525	506/972,491	459/1,268,361	380/1,118,944	330/1,073,106	110/715,691	
2y-	IR(95%CI)	40.2(36.3–44.5)	29.7(26.1–33.9)	32.0(28.5–36.0)	22.9(20.4–25.7)	18.6(16.3–21.3)	19.6(17.1–22.5)	10.3(8.3–12.7)	34%
	Cases/PY	379/942,888	226/759,795	281/876,966	288/1,255,921	215/1,153,571	208/1,059,401	84/818,271	
3y-	IR(95%CI)	35.8(31.9–40.3)	26.3(22.9–30.2)	23.9(20.8–27.6)	20.1(17.7–22.7)	14.1(12.1–16.4)	16.8(14.4–19.4)	10.3(8.3–12.7)	36%
	Cases/PY	280/781,788	202/767,820	189/789,495	246/1,225,646	168/1,192,088	176/1,050,728	86/834,530	
4y-	IR(95%CI)	29.0(25.1–33.6)	23.6(20.4–27.3)	20.1(17.1–23.6)	17.5(15.2–20.1)	13.7(11.8–15.9)	13.3(11.3–15.7)	9.0(7.2–11.3)	44%
	Cases/PY	180/620,852	184/779,542	150/745,528	203/1,161,046	168/1,226,201	141/1,059,744	74/821,630	
5y-	IR(95%CI)	28.8(24.3–34.3)	19.9(17.0–23.2)	17.8(15.0–21.1)	12.6(10.7–15.0)	12.8(11.0–14.9)	11.4(9.5–13.6)	9.1(7.3–11.5)	43%
	Cases/PY	130/450,662	158/795,685	131/737,412	134/1,061,312	160/1,250,384	122/1,073,190	74/810,772	
6y-	IR(95%CI)	24.3(19.1–30.9)	19.7(16.9–23.0)	16.5(13.8–19.7)	15.6(13.3–18.3)	13.4(11.5–15.6)	10.5(8.8–12.6)	7.6(5.9–9.8)	47%
	Cases/PY	66/271,722	161/816,483	122/740,886	150/961,877	168/1,254,561	116/1,102,137	61/802,833	
7y-	IR(95%CI)	17.3(10.6–28.3)	20.0(17.1–23.2)	13.2(10.9–16.1)	15.6(13.2–18.5)	10.7(9.0–12.7)	9.8(8.1–11.8)	8.7(6.8–11.0)	51%
	Cases/PY	16/92,389	167/836,764	99/748,405	135/865,857	133/1,241,664	111/1,135,657	69/797,367	
8y-	IR(95%CI)	-	18.0(15.2–21.2)	15.4(12.8–18.5)	12.8(10.5–15.5)	9.8(8.2–11.7)	10.8(9.1–12.8)	8.3(6.5–10.5)	40%
	Cases/PY	-	139/773,342	117/759,844	100/782,709	119/1,217,165	127/1,178,527	66/797,576	
9y-	IR(95%CI)	-	18.1(15.1–21.8)	15.7(13.2–18.8)	13.6(11.2–16.5)	11.5(9.7–13.6)	10.3(8.6–12.2)	7.8(6.1–10.0)	43%
	Cases/PY	-	111/612,234	121/768,540	100/735,772	132/1,148,569	124/1,208,932	63/805,964	
<1y	IR(95%CI)	895.2(878.9–911.8)	666.6(648.4–685.3)	622.5(607.8–637.6)	488.8(476.7–501.1)	385.8(374.4–397.7)	315.2(304.8–325.9)	117.7(108.8–127.3)	53%
	Cases/PY	11,340/1,266,785	4,995/749,332	6,688/1,074,348	6,188/1,266,025	4,210/1,091,121	3,429/1,088,019	622/528,390	
1-4y	IR(95%CI)	45.6(43.4–47.9)	32.4(30.4–34.4)	33.3(31.4–35.3)	24.4(23.0–25.8)	19.8(18.6–21.2)	20.2(18.8–21.5)	11.1(10.0–12.3)	38%
	Cases/PY	1,573/3,449,109	990/3,058,682	1,126/3,384,480	1,196/4,910,974	931/4,690,805	855/4,242,980	354/3,190,121	
5-9y	IR(95%CI)	26.0(22.7–29.8)	19.2(17.9–20.6)	15.7(14.5–17.0)	14.0(13.0–15.2)	11.6(10.8–12.5)	10.5(9.7–11.4)	8.3(7.5–9.2)	45%
	Cases/PY	212/814,773	736/3,834,508	590/3,755,087	619/4,407,527	712/6,112,342	600/5,698,443	333/4,014,512	

^a^d% = (rate_1981-1985_-rate_2001-2005_)/rate_1981-1985_; Cases: the number of deaths; PY: person years.

### Mortality due to neoplasms

**Fig 3:**
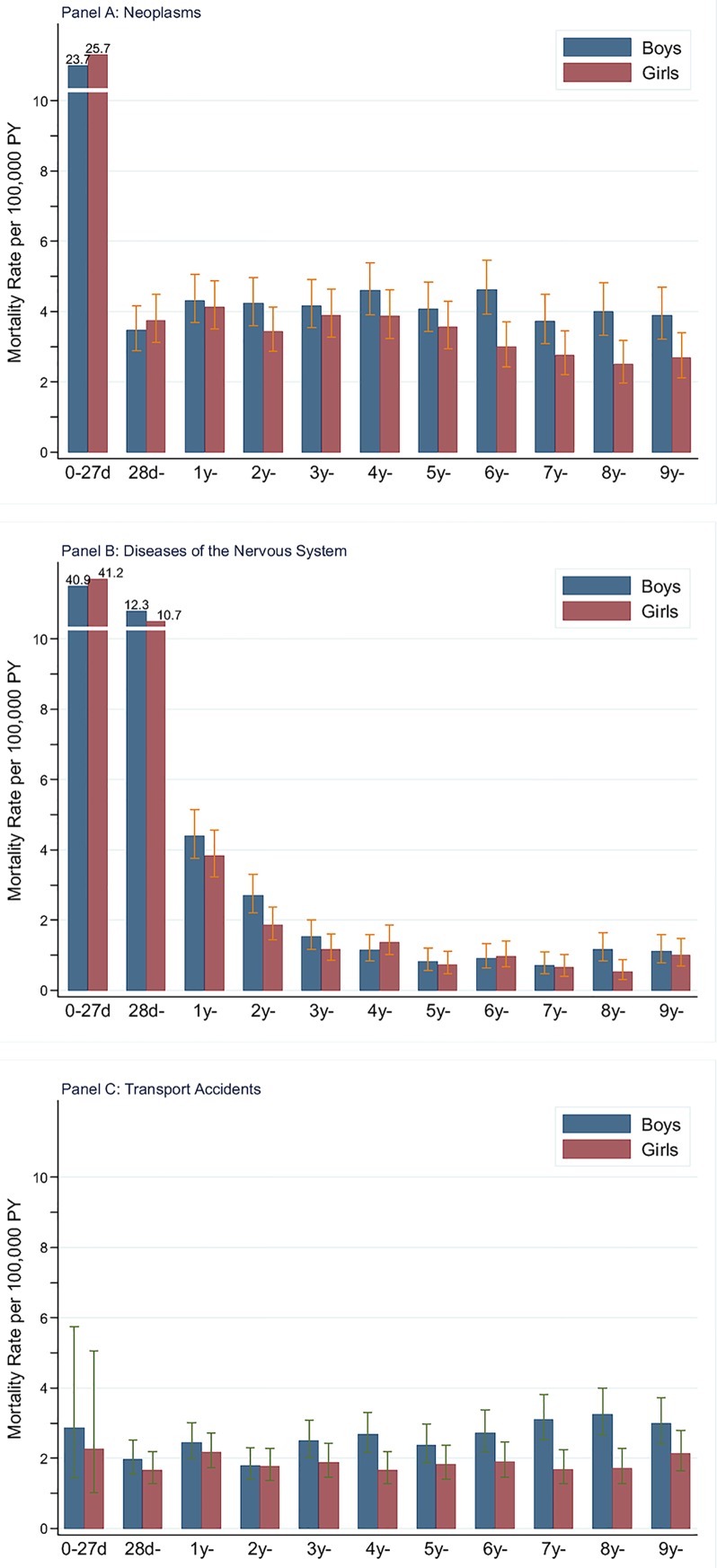
Mortality rates of the three most frequent causes in children aged 0–9 years over age and gender (28d: 28–364 Days).

**Fig 4:**
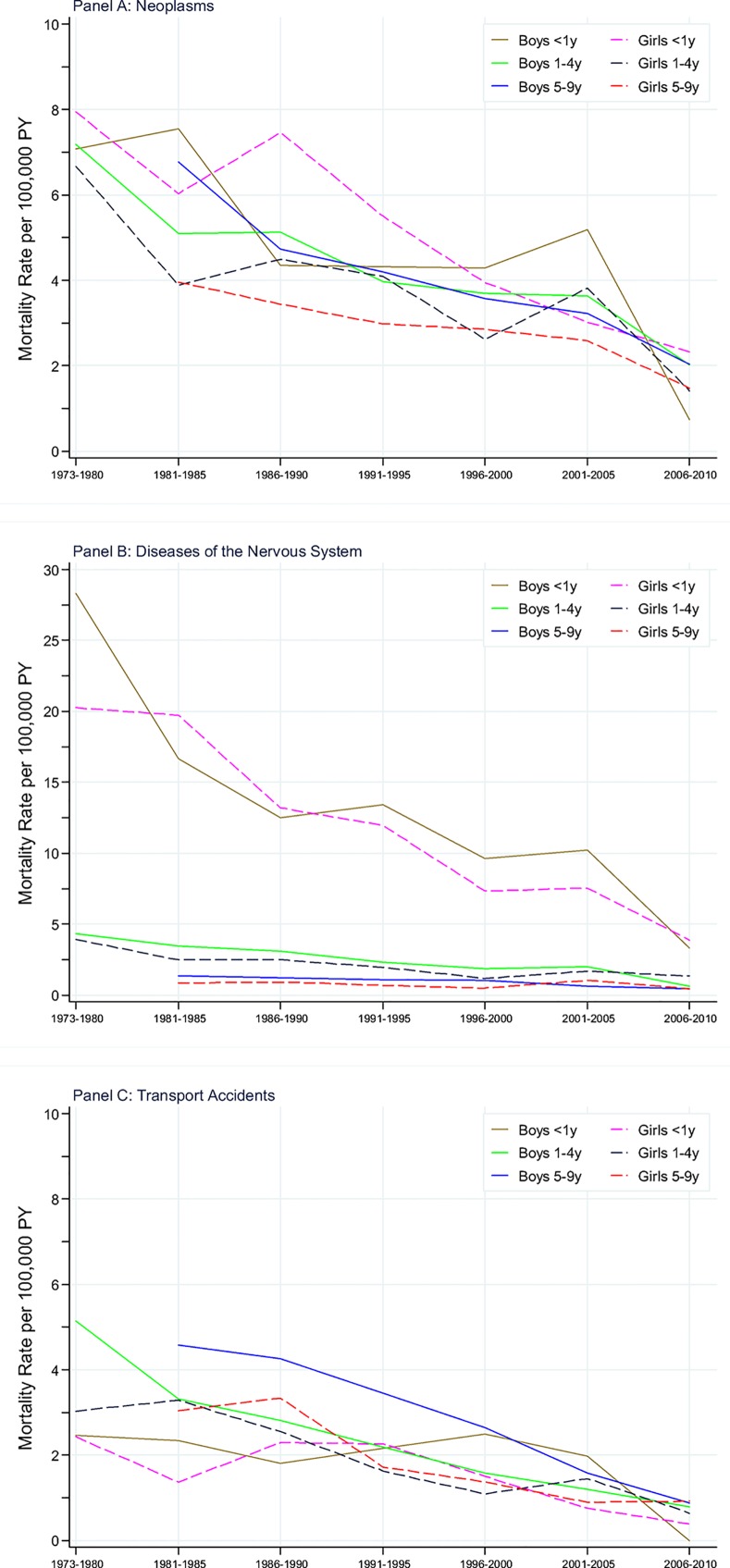
Mortality rates of the three most frequent causes in children aged 0–9 years over calendar periods, aggregated age groups and gender.

Neoplasm was the most frequent cause of death after 1 year of age and cumulative mortality before 10 years was 39 (95% CI: 37–40) per 100,000, with boys of 42 (95%CI: 40–45) per 100,000 and girls of 35 (95%CI: 33–37) per 100,000. Both genders had higher mortality at 0–27 days than in other ages (Fig 3A). Boys had lower mortality than girls with a RR_m/f_ of 0.92 (95%CI: 0.75–1.13) before 1 year of age. After 1 year of age, boys had higher mortality rate than girls with the RR_m/f_ from 1.04 to 1.60. We found no obvious change in patterns of mortality rate among age-groups. When taking length of time into consideration, mortality rate decreased by 42% in boys and 25% in girls. Mortality rate showed an overall downward directionality with calendar periods in aggregated age-groups with obvious fluctuations among calendar periods (Fig 4A; S5 Table). Particularly, boys less than 1 year and girls aged 1–4 years reached the second peak in 2001–2005. During 1981–1985, boys had higher mortality rate than girls. Mortality rate was 6.18 per 100,000 person years in boys and 4.13 per 100,000 person years in girls. During 2001–2005, boys still had higher mortality than girls except for children aged 1–4 years. Mortality rates in boys and girls were 3.57 and 3.11 per 100,000 person years, respectively. RR_m/f_ in children less than 10 years decreased from 1.49 (95%CI: 1.22–1.83) to 1.15 (95%CI: 0.94–1.41) with a reduction in children aged 5–9 years from 1.71 (95%CI: 1.27–2.27) to 1.24 (95%CI: 0.91–1.69) (S4 Table). Leukemia (31.9%), brain and other central nervous system (CNS) tumours (31.6%) were the most common cause of death by neoplasms in children before 10 years. Mortality rate due to leukemia, brain and CNS tumours decreased by 50% (66% in boys, 23% in girls), and 36% (38% in boys, 34% in girls) from 1981–1985 to 2001–2005, respectively.

### Mortality due to diseases of the nervous system

Disease of the nervous system was the most frequent cause of death in children of both 1 year and 1–9 years of age and cumulative mortality before 10 years was 27 (95%CI: 25–28) per 100,000, with boys of 29 (95%CI: 27–31) per 100,000 and girls of 25 (95%CI: 23–26) per 100,000. The highest risk was obtained among children younger than 1 year and 52% of deaths occurred before 1 year of age. Mortality rates decreased with age in the early ages and kept relatively stable after 3 years of age (Fig 3B). Boys had a slightly higher overall mortality risk than girls (RR_m/f_: 1.18, 95%CI: 1.07–1.29), but a pronounced gender difference was not observed in separated age groups. When taking length of follow-up time into account, mortality in children less than 1 year had declined remarkably. In contrast, the decline in mortality rate after 1 year of age was modest. At 5–9 years of age, mortality rate remained quite stable around 1.00 per 100,000 person years across the entire follow-up period.

In most calendar periods, mortality rates in boys were slightly higher than that in girls (Fig 4B; S6 Table). During 1981–1985, boys had higher mortality rate than girls, except for infant mortality. Mortality rate was 3.70 per 100,000 person years in boys and 3.36 per 100,000 person years in girls, giving a RR_m/f_ of 1.10(95%CI: 0.87–1.40). Infant mortality rate (<1 year) decreased dramatically over calendar periods, and from 1 year of age mortality rate was relatively stable across calendar periods (Fig 4B). During 2001–2005, boys had higher mortality rate than girls except for children aged 5–9 years (S4 Table). Mortality rate was 2.11 per 100,000 person years in boys and 1.93 per 100,000 person years in girls, giving a RR_m/f_ of 1.09(95%CI: 0.84–1.42).

Among the diseases of the nervous system, meningitis is the first leading cause of death (28.5%), followed by cerebral palsy (12.7%) in children before 10 years. More than 64% of deaths by meningitis and 44% of deaths by cerebral palsy occurred in infancy. Mortality rate due to meningitis and cerebral palsy in children under 10 years decreased by 65% (68% in boys, 20% in girls), and 68% (76% in boys, 60% in girls) from 1981–1985 to 2001–2005.

### Mortality due to transport accidents

Transport accidents was the most frequent cause of death after 1 year of age and cumulative mortality before 10 years was 22 (95% CI: 21–23) per 100,000, with boys of 26 (95% CI: 24–28) per 100,000 and girls of 18 (95% CI: 17–20) per 100,000. Mortality rate in boys increased with age while those in girls differed slightly between ages (Fig 3C). Boys had higher mortality rate than girls in all age groups and calendar periods except for children aged 1–4 year in 2001–2005 (Fig 4C; S4 Table and S7 Table). During 1981–1985, mortality rates in boys and girls were 3.85 and 2.98 per 100,000 person years, respectively. Mortality rates decreased with calendar periods in four aggregated age groups. However, among girls mortality showed early growth and later decline from 1986–1990 onwards. During 2001–2005, mortality rates in boys and girls were 1.47 and 1.10 per 100,000 person years, respectively. RR_m/f_ in children less than 10 years increased from 1.29 (95%CI: 1.01–1.65) to 1.34 (95%CI: 0.96–1.87) with a large increase in age group of 5–9 years from 1.50 (95%CI: 1.08–2.09) to 1.75 (95%CI: 1.08–2.85). Compared with the other three groups (including boys aged 1–4 years, girls aged 1–4 years and girls aged 5–9 years), boys aged 5–9 years had higher mortality rate in all calendar period. Because of the very small number of deaths before 1 year of age in both genders, mortality rate in infancy was not compared with those in other age groups.

## Discussion

This study presents an overview of mortality directionality in children aged 0–9 years in combined data of three Nordic countries. In this study, which to our knowledge is the first study examining mortality over age at 1-year interval, we found that overall mortality rates decreased with age before 5 years and were relatively stable between 5 and 9 years. Mortality rate in boys was higher than that in girls at each age and the gender differences were more pronounced in children between the ages of 5–9 years. From 1981–1985 to 2001–2005, all-cause mortality rates were reduced by between 34% and 62% at different ages. Mortality rate ratio between boys and girls aged 0–9 years decreased with a pronounced decline in aged 5–9 years. Mortality in infancy (<1 year) making up 70% of the total deaths, still greatly exceeded that of all other ages in the recent years of 2006–2010. Most of death in infancy was caused by certain conditions originating in the prenatal periods, congenital malformations, sudden infant death syndrome, and unknown or unspecified causes. After 1 year of year, neoplasms were the most frequent cause of death, followed by diseases of the nervous system and transport accidents. These three most important causes of death declined over time, both in boys and girls. Mortality from neoplasms was the highest in each age group except for infants among the three leading causes of death, and half of deaths from diseases of the nervous system occurred in infancy. Mortality due to transport accidents increased with age and boys aged 5–9 years had higher risk compared to boys and girls in any other age groups.

### All-cause mortality

Our finding of a reduction in mortality in children aged 0–9 years in the past decades is consistent with previous studies from high-income countries [5, 9–12]. Unlike previous studies [5, 9, 11, 12], we had individual level data to compute person years and mortality rate in our study can be expressed as deaths per 100,000 person years. We found that mortality rate decreased with age and was relatively stable between 5 to 9 years. Boys had higher mortality than girls at each age and the difference between genders was more pronounced between 5 to 9 years.

### Infant mortality

Infant mortality rate has decreased by more than 50% from 1981–1985 to 2001–2005. Preterm birth complications are the leading cause of death in neonates and the second common cause of death in children younger than 5 years[13]. Surviving preterm infants are at increased long-term risk of neurodevelopmental impairments and respiratory and gastrointestinal complications [14]. Interventions, advances in technologies and the collaborative efforts of obstetricians and neonatologists have not only increased the survival rate of preterm infants and prematurity-related neonatal morbidity, but may possibly also have decreased the risk in later life [15–17]. Studies in Finland and Sweden also showed that advancing neonatal intensive care is associated with decreased death of extremely premature infants [18, 19]. The national Guidelines for Perinatal Care in the USA recommend that all infants <32 weeks gestation and infants with very low birth weight are born in subspecialty perinatal centers. As infants still have much higher mortality risk than other ages, special attention and continued effort should be focused on better survival of preterm births. The reduction in mortality from congenital malformation is consistent with the result from the previous study in Canada, which might be the results of improved prenatal diagnose with subsequent termination of affected pregnancies and improved surgical repair [20]. The decrease in mortality from sudden infant death syndrome might be due in part to diagnostic shift and better ascertainment of deaths previously registered as sudden infant death syndrome[21]. SIDS cannot be prevented completely but a better understanding of the circumstances and events associated with sleep-related infant deaths may help reduce the risks for SIDS and other sleep-related causes of infant death.

### Gender difference

Our data showed that boys had higher mortality than girls in the total study period, which was in line with the results from the studies in Nordic countries and from the UK [10, 11, 22, 23]. Few studies have reported mortality difference between genders over time in children and limited studies focused on these differences by country, parental education or age [10, 24]. Our data showed that gender mortality differences in children decreased with time in three Nordic countries. Generally, there is currently no accepted explanation for why female mortality is lower than male [25]. Gender differences in mortality could be influenced by biological, genetic, social, cultural, environmental and behavioral factors [26], which need further investigation for preventive measures.

### Mortality due to neoplasms

Mortality rate due to neoplasms in both genders decreased dramatically with time. The ACCIS project in Europe [27] reported that 5-year survival in children (0–14 years) with cancer has improved remarkably since the 1970s. Surveillance research from American Cancer Society [28] reported death rates for all childhood cancers (0–14 years) decreased steadily from 1975 to 2010, and mortality rates are lower in girls than in boys in US. In our study, the difference of neoplasms mortality between genders was reduced with time. Over recent decades, survival from childhood cancer has improved dramatically because of improvements in chemotherapy and radiation treatment protocols, better diagnosis, and risk classification and improved supportive care [29]. In Nordic countries, some specific programs may explain the reduction of mortality in children. The Nordic Society of Paediatric Haematology and Oncology (NOPHO) started a registration in five Nordic countries in 1981 to create uniform diagnostic, treatment, and clinical follow-up procedures for childhood cancers [30]. In addition, the Nordic Cancer Union, a collaborative body for cancer societies in the Nordic countries, has a wide range of cooperative activities to provide more effective prevention of cancer and to advance the diagnostic procedures and treatment strategies for cancer patients. As a corollary of these initiatives, the Nordic cooperation has led to a gradual improvement in the treatment and prognosis of childhood cancers. For acute lymphoblastic leukemia, the most common cancer in children, the overall event-free survival at 5 years has increased from 57% in the early 1980s to 78% during the 1990s in five Nordic countries [31]. For children with acute myeloid leukaemia, the 5-year event-free survival and overall survival increased from 29% and 38% in July 1984, to 48% and 65% in December 2001, respectively [32]. The report from National Cancer Institute[33] suggested that changes in diagnostic and improvement in treatment procedures since the mid-1970s have resulted in improved survival rates for patients with brain tumours. Our studies also showed that mortality rate by brain and other central nervous system tumours decreased over the past years. Although our study demonstrated mortality by neoplasms had an overall decreased directionality with calendar periods, the obvious peaks were observed in boys less than 1 year and girls between 1 and 4 years in 2001–2005.

### Mortality due to diseases of the nervous system

Our study showed that half of death due to diseases of the nervous system occurred in infancy (<1 year). Over calendar period, mortality rates were decreased by 43% in boys and 42% in girls. Holt et al [34] previously reported that neonatal meningitis in England and Wales appeared to be falling. The progress can mainly be ascribed to successful vaccination programmes (against Haemophilus influenzae type B, Streptococcus pneumonia and N. meningitis), advances in diagnosis and treatment [35–37]. The conjugated Haemophilus influenzae type B vaccine, which was administered routinely to all infants in Denmark in 1993, in Sweden in 1992 and in Finland in 1990, may explain the sharp decrease in mortality rate in infancy from 1991–1995 to 1996–2000 [37–39]. A US study [40] reported a decreased hospitalization for pneumococcal meningitis among children 0–4 years after implementation of routine childhood vaccination with a 7-valent pneumococcal conjugate vaccine (PCV7). PCV7 has been included in the childhood vaccination programme since 2007 in Denmark [37] and since 2008[41] in Sweden, which corresponded with the timing of the decrease from 2001–2005 to 2006–2010. A cohort study in North Carolina showed that mortality of very low birth weight infants by cerebral palsy decreased from 36.8% to 13.8% from 1982–1984 to 1992–1994 and the better survival may be owing to improvements in obstetric care [42].

### Mortality due to transport accidents

We observed that mortality due to transport accident was higher in boys than in girls at each age. In boys, mortality rates increased with age, reaching a relatively high level at 7 to 9 years. In contrast, mortality rates in girls remained quite stable. A study from 50 countries [43] identified transport injuries as the dominant causes of injury-related deaths in children aged 1–9 years. The study also reported an early increase and later decline, which were probably associated with the introduction of road safety measures. We also observed this pattern in girls but not in boys. The data from Scotland [44] reported that road-traffic injuries were the leading causes of injury death in children aged 5–9 years despite declining mortality rates from 2002 to 2006, but the difference between genders was not provided. A probable explanation is that children aged 8 to 14 are often easily distracted and therefore more prone to traffic accidents [45]. As boys are more likely to engage in risky behaviours than girls, their propensity towards risk-taking behaviours makes boys have a higher likelihood of being involved in fatal traffic accidents than girls [45, 46]. When taking length of follow-up time into account, mortality rate has decreased by almost 62% in boys and 63% in girls from 1981–1985 to 2001–2005. Our results are in line with the study examining childhood deaths and injuries among children aged 0–14 years in Finland [4]. Our study showed that mortality reduction of transport accidents exceeded that of the other two causes of death (neoplasms and disease of the nervous system), for both boys and girls. Dedicated education campaigns to road safety, improved traffic safety and mandatory use of safety belt, and child restraints may play an important role in such a mortality reduction [45]. Also, improvement in trauma care may also contribute to the reduction [4]. Our findings suggest the effectiveness of those preventive measures and may further reduce deaths due to preventable causes.

### Strengths and limitations

Our study has a number of strengths. We combined nationwide data from three Nordic countries in which have complete individual level register data on population and their deaths. The individual level data over two decades permitted us to examine mortality directionality across a different time periods as well as major groups of cause of death. Even with the small number of death in children aged from 1 to 9 years, especially from 5 to 9 years, the combined data from the three countries still provides the possibility to examine the mortality over age in 1- year interval.

Our findings should also be interpreted in the light of limitations. First, we cannot rule out the possibility that changes in long-term directionality in cause-specific mortality are related to coding changes between and within ICD revisions [47]. Second, the total number of death was relatively small in some age groups, and our study may not have enough statistical power to detect gender differences between within age groups.

## Conclusion

We observed decreased mortality in children at 0–9 years in the three Nordic countries during the past decades. Mortality rates showed overall downward directionality with age and calendar time, and suggests further possibilities for improving survival in children. Infants, particularly in neonates, have much higher mortality than children aged 1–9 years. Mortality rate in boys was higher than in girls and gender difference on mortality declined with calendar time. Although advances in treatment have improved the survival rate of children with cancer, neoplasms is still the most frequent cause of death in children aged 1–9 years in the three Nordic countries and mortality was the highest in each age group except for infants. Mortality due to transport accidents, one of the most common causes of death in children, increased with age and was highest in boys aged 5–9 years. Our results suggest the importance of further study on mortality by causes of neoplasms, and causes of transport accidents—especially in children aged 5–9 years.

## Ethics Statement

The data collection was approved by the Data Protection Agency and Research Ethics Committee of the Central Region in Denmark, the Research Ethics Committee (EPN) at Karolinska Institute in Sweden, the National Institute for Health and Welfare (THL) in Finland and Statistics Finland. No informed consent from participants is required as the register-based study is based on secondary data for which participants’ information was anonymized and de-identified.

## Supporting Information

S1 Table A) The number of deaths by causes of death. B) All-cause mortality rate by age, gender and calendar period. (XLSX)

S2 Table Mortality rate due to natural death by age, gender and calendar period. (XLSX)

S3 Table Mortality rate due to unnatural death by age, gender and calendar period. (XLSX)

S4 Table Mortality rate ration of boys to girls between 1981–1995 and 2001–2005. (XLSX)

S5 Table Mortality rate due to neoplasms by age, gender and calendar period. (XLSX)

S6 Table Mortality rate due to diseases of the nervous system by age, gender and calendar period. (XLSX)

S7 Table Mortality rate due to diseases of transport accidents by age, gender and calendar period. (XLSX)

## References

[1] UNICEF(2013). Levels & Trends in Child Mortality Report 2013. Available: http://www.childinfo.org/files/Child_Mortality_Report_2013.pdf. Accessed 25 August 2014.

[2] UNICEF(2013). Committing to Child Survival: A Promise Renewed, Progress Report 2013. Available: http://www.unicef.org/publications/index_70354.html. Accessed 25 August 2014.

[3] UNICEF(2011). The State of the World's Children 2011 Adolescence: an Age of Opportunity. Available: http://www.unicef.org/sowc2011/. Accessed 25 August 2014.

[4] Parkkari J, Kannus P, Niemi S, Koskinen S, Palvanen M, Vuori I, et al Childhood deaths and injuries in Finland in 1971–1995. International journal of epidemiology. 2000;29(3):516–23. 10869325

[5] Lantto M, Renko M, Uhari M. Trends in childhood mortality from 1969 to 2004 in Finland. Acta paediatrica. 2008;97(8):1024–9. Epub 2008/05/14. 10.1111/j.1651-2227.2008.00856.x . 18474069

[6] Li J, Vestergaard M, Obel C, Cnattingus S, Gissler M, Olsen J. Cohort profile: the Nordic Perinatal Bereavement Cohort. International journal of epidemiology. 2011;40(5):1161–7. 10.1093/ije/dyq127 . 20675718

[7] Official Statistics of Finland (OSF): Causes of death [e-publication]. ISSN = 1799–5078. 2012, Appendix table 1b. Deaths by underlying cause of death and by age in 2012, males. Helsinki: Statistics Finland [referred: 10.12.2015]. Access method: http://www.stat.fi/til/ksyyt/2012/ksyyt_2012_2013-12-30_tau_002_en.html.

[8] Rothman KJ, Greenland S, Lash TL. Modern epidemiology: Lippincott Williams & Wilkins; 2008.

[9] Lantto M, Renko M, Uhari M. Regional differences in postneonatal childhood mortality in Finland, 1985–2004. Acta paediatrica. 2014 10.1111/apa.12853 . 25378189

[10] Gissler M, Rahkonen O, Mortensen L, Arntzen A, Cnattingius S, Nybo Andersen AM, et al Sex differences in child and adolescent mortality in the Nordic countries, 1981–2000. Scandinavian journal of public health. 2009;37(4):340–6. 10.1177/1403494809103905 . 19286748

[11] Sidebotham P, Fraser J, Covington T, Freemantle J, Petrou S, Pulikottil-Jacob R, et al Understanding why children die in high-income countries. Lancet. 2014;384(9946):915–27. 10.1016/S0140-6736(14)60581-X . 25209491

[12] Viner RM, Hargreaves DS, Coffey C, Patton GC, Wolfe I. Deaths in young people aged 0–24 years in the UK compared with the EU15+ countries, 1970–2008: analysis of the WHO Mortality Database. Lancet. 2014;384(9946):880–92. 10.1016/S0140-6736(14)60485-2 . 24929452

[13] Liu L, Johnson HL, Cousens S, Perin J, Scott S, Lawn JE, et al Global, regional, and national causes of child mortality: an updated systematic analysis for 2010 with time trends since 2000. Lancet. 2012;379(9832):2151–61. 10.1016/S0140-6736(12)60560-1 . 22579125

[14] Goldenberg RL, Culhane JF, Iams JD, Romero R. Epidemiology and causes of preterm birth. Lancet. 2008;371(9606):75–84. 10.1016/S0140-6736(08)60074-4 . 18177778 PMC7134569

[15] Richardson DK, Gray JE, Gortmaker SL, Goldmann DA, Pursley DM, McCormick MC. Declining severity adjusted mortality: evidence of improving neonatal intensive care. Pediatrics. 1998;102(4 Pt 1):893–9. . 9755261 10.1542/peds.102.4.893

[16] Saigal S, Doyle LW. An overview of mortality and sequelae of preterm birth from infancy to adulthood. The Lancet. 2008;371(9608):261–9. 10.1016/s0140-6736(08)60136-1 18207020

[17] Iams JD, Romero R, Culhane JF, Goldenberg RL. Primary, secondary, and tertiary interventions to reduce the morbidity and mortality of preterm birth. The Lancet. 2008;371(9607):164–75. 10.1016/s0140-6736(08)60108-7 18191687

[18] Rautava L, Lehtonen L, Peltola M, Korvenranta E, Korvenranta H, Linna M, et al The effect of birth in secondary- or tertiary-level hospitals in Finland on mortality in very preterm infants: A birth-register study. Pediatrics. 2007;119(1):E257–E63. 10.1542/peds.2006-1964 . 17200251

[19] Group E, Fellman V, Hellstrom-Westas L, Norman M, Westgren M, Kallen K, et al One-year survival of extremely preterm infants after active perinatal care in Sweden. JAMA: the journal of the American Medical Association. 2009;301(21):2225–33. 10.1001/jama.2009.771 . 19491184

[20] Wen SW, Liu S, Joseph KS, Rouleau J, Allen A. Patterns of infant mortality caused by major congenital anomalies. Teratology. 2000;61(5):342–6. 10.1002/(SICI)1096-9926(200005)61:5<342::AID-TERA5>3.0.CO;2-7 . 10777829

[21] Sidebotham P, Fraser J, Fleming P, Ward-Platt M, Hain R. Patterns of child death in England and Wales. Lancet. 2014;384(9946):904–14. 10.1016/S0140-6736(13)61090-9 . 25209490

[22] Gissler M, Jarvelin MR, Louhiala P, Hemminki E. Boys have more health problems in childhood than girls: follow-up of the 1987 Finnish birth cohort. Acta paediatrica. 1999;88(3):310–4. . 10229043 10.1080/08035259950170088

[23] Mortality Statistics: General, England and Wales (Series DH1: discontinued)—No. 38, 2005.

[24] Gissler M, Rahkonen O, Mortensen L, Arntzen A, Cnattingius S, Nybo Andersen AM, et al Sex differences in child and adolescent mortality by parental education in the Nordic countries. Journal of epidemiology and community health. 2012;66(1):57–63. 10.1136/jech.2009.093153 . 20974838

[25] Mage DT, Donner M. Female resistance to hypoxia: Does it explain the sex difference in mortality rates? J Womens Health. 2006;15(6):786–94. 10.1089/jwh.2006.15.786 . 16910910

[26] Kalben BB. Why men die younger: causes of mortality differences by sex. North American Actuarial Journal. 2000;4(4):83–111.

[27] Steliarova-Foucher E, Stiller C, Kaatsch P, Berrino F, Coebergh JW, Lacour B, et al Geographical patterns and time trends of cancer incidence and survival among children and adolescents in Europe since the 1970s (the ACCIS project): an epidemiological study. Lancet. 2004;364(9451):2097–105. 10.1016/S0140-6736(04)17550-8 . 15589307

[28] American Cancer Society. Cancer Facts & Figures 2014. Atlanta, Ga: American Cancer Society; 2014.

[29] Davies SM. Subsequent malignant neoplasms in survivors of childhood cancer: Childhood Cancer Survivor Study (CCSS) studies. Pediatr Blood Cancer. 2007;48(7):727–30. 10.1002/pbc.21113 . 17243132

[30] Hjalgrim LL, Rostgaard K, Schmiegelow K, Soderhall S, Kolmannskog S, Vettenranta K, et al Age- and sex-specific incidence of childhood leukemia by immunophenotype in the Nordic countries. J Natl Cancer Inst. 2003;95(20):1539–44. . 14559876 10.1093/jnci/djg064

[31] Gustafsson G, Schmiegelow K, Forestier E, Clausen N, Glomstein A, Jonmundsson G, et al Improving outcome through two decades in childhood ALL in the Nordic countries: the impact of high-dose methotrexate in the reduction of CNS irradiation. Leukemia. 2000;14(12):2267–75. 10.1038/sj.leu.2401961 . 11187918

[32] Lie SO, Abrahamsson J, Clausen N, Forestier E, Hasle H, Hovi L, et al Long-term results in children with AML: NOPHO-AML Study Group—report of three consecutive trials. Leukemia. 2005;19(12):2090–100. 10.1038/sj.leu.2403962 . 16304571

[33] Davis FG, Freels S, Grutsch J, Barlas S, Brem S. Survival rates in patients with primary malignant brain tumors stratified by patient age and tumor histological type: an analysis based on Surveillance, Epidemiology, and End Results (SEER) data, 1973–1991. J Neurosurg. 1998;88(1):1–10. 10.3171/jns.1998.88.1.0001 . 9420066

[34] Holt DE, Halket S, de Louvois J, Harvey D. Neonatal meningitis in England and Wales: 10 years on. Archives of disease in childhood Fetal and neonatal edition. 2001;84(2):F85–9. 11207221 10.1136/fn.84.2.F85 PMC1721232

[35] Saez-Llorens X, McCracken GH Jr. Bacterial meningitis in children. Lancet. 2003;361(9375):2139–48. 10.1016/S0140-6736(03)13693-8 . 12826449

[36] Peltola H, Kilpi T, Anttila M. Rapid Disappearance of Haemophilus-Influenzae Type-B Meningitis after Routine Childhood Immunization with Conjugate Vaccines. Lancet. 1992;340(8819):592–4. 10.1016/0140-6736(92)92117-X . 1355165

[37] Howitz M, Hartvig Christiansen A, Harboe ZB, Molbak K. Surveillance of bacterial meningitis in children under 2 y of age in Denmark, 1997–2006. Scandinavian journal of infectious diseases. 2008;40(11–12):881–7. 10.1080/00365540802325914 . 18720256

[38] Garpenholt O, Silfverdal SA, Hugosson S, Fredlund H, Bodin L, Romanus V, et al The impact of Haemophilus influenzae type b vaccination in Sweden. Scandinavian journal of infectious diseases. 1996;28(2):165–9. . 8792484 10.3109/00365549609049069

[39] Peltola H, Kilpi T, Anttila M. Rapid disappearance of Haemophilus influenzae type b meningitis after routine childhood immunisation with conjugate vaccines. The Lancet. 1992;340(8819):592–4. 10.1016/0140-6736(92)92117-x 1355165

[40] Tsai CJ, Griffin MR, Nuorti JP, Grijalva CG. Changing epidemiology of pneumococcal meningitis after the introduction of pneumococcal conjugate vaccine in the United States. Clinical Infectious Diseases. 2008;46(11):1664–72. 10.1086/587897 18433334 PMC4822508

[41] Silfverdal SA, Berg S, Hemlin C, Jokinen I. The cost-burden of paediatric pneumococcal disease in Sweden and the potential cost-effectiveness of prevention using 7-valent pneumococcal vaccine. Vaccine. 2009;27(10):1601–8. 10.1016/j.vaccine.2008.12.033 19146905

[42] O'Shea TM, Preisser JS, Klinepeter KL, Dillard RG. Trends in mortality and cerebral palsy in a geographically based cohort of very low birth weight neonates born between 1982 to 1994. Pediatrics. 1998;101(4):642–7. 10.1542/peds.101.4.642 . 9521949

[43] Viner RM, Coffey C, Mathers C, Bloem P, Costello A, Santelli J, et al 50-year mortality trends in children and young people: a study of 50 low-income, middle-income, and high-income countries. Lancet. 2011;377(9772):1162–74. 10.1016/S0140-6736(11)60106-2 . 21450338

[44] Pearson J, Stone DH. Pattern of injury mortality by age-group in children aged 0–14 years in Scotland, 2002–2006, and its implications for prevention. BMC pediatrics. 2009;9:26 10.1186/1471-2431-9-26 19351386 PMC2674042

[45] DaCoTA (2012) Children in road traffic, Deliverable 4.8c of the EC FP7 project DaCoTA.

[46] Harris CR, Jenkins M, Glaser D. Gender differences in risk assessment: Why do women take fewer risks than men? Judgm Decis Mak. 2006;1(1):48–63. .

[47] Janssen F, Kunst AE. ICD coding changes and discontinuities in trends in cause-specific mortality in six European countries, 1950–99. Bulletin of the World Health Organization. 2004;82(12):904–13. doi: /S0042-96862004001200006. 15654404 PMC2623106

